# Predictors of Use of Preventative Health Services for People with Disabilities in Taiwan

**DOI:** 10.3390/ijerph18041661

**Published:** 2021-02-09

**Authors:** Tzu-Ying Chiu

**Affiliations:** Department of Health and Welfare, College of City Management, University of Taipei, Taipei City 11153, Taiwan; yin827@gmail.com; Tel.: +886-2871-8288

**Keywords:** activity and participation, WHODAS 2.0, preventive health services

## Abstract

People with disabilities display less use of preventive health services, such as health examinations, flu vaccinations, Pap smears and breast screening, but evidence has shown that preventive health services can detect or even prevent serious diseases and medical problems. Therefore, identifying the factors associated with the use of preventive health services is important for people with disabilities. This study examined the use of preventive health services by people with disabilities and identified other associated factors for people with disabilities. The research used social demographics and the World Health Organization Disability Assessment Schedule 2.0 (WHODAS 2.0) 12 items to measure activity and participation (AP) and other factors; there were 742 people with disabilities recruited with stratified proportional sampling. The data were collected through face-to-face interviews. The findings revealed that the common types of preventive services accessed by people with disabilities were health examinations and flu vaccinations; most of them had only used one preventive health service in the past year. The factors of having caregivers of spouses (OR = 1.74), perceived good health (OR = 1.26), and less limitation of AP (OR = 0.99) were significantly associated with the use of preventive services (*p* < 0.01). The study found a significant association between having children as caregivers and the non-use of Pap smears and breast screening services among women, providing valuable evidence for the distribution of the use of preventive health services for people with disabilities. Furthermore, the study highlighted the present status of disparities in the use of preventive services for people with disabilities and should encourage a boost in the adjustment of the medical environment and service resource allocation by the Taiwanese government for people with disabilities.

## 1. Background

The population of people with disabilities is increasing all over the world. To maintain a good quality of life as one ages, the Sustainable Development Goals (SDGs) and World Health Organization (WHO) Global Disability Action Plan suggest ways to pursue a healthy life and well-being, including strategies for reducing inequalities within countries and improving access to health services and programs for people with disabilities [1,2]. The WHO stated that functional ability and environmental factors are key to achieving good health [3].

Many indicators can be used to evaluate people’s health functioning problems and limitations. However, the majority of the evaluation tools are based on medical models [3], which evaluate impairments in body structure and the severity of disability and focus less on the difficulties experienced by people with disabilities after the occurrence of an impairment. Nevertheless, one indicator that focuses on the last is activity and participation (AP), which is a comprehensive concept that refers to the individual execution of a task and the manner in which such tasks are involved in daily life. The concept of AP was proposed by the International Classification of Functioning, Disability and Health (ICF), which provides a standard language and multidimensional perspective for evaluating health as well as an integrated biopsychosocial model of disability and functioning for individuals and the entire population [3]. The classification system was operationalized through the WHO Disability Assessment Schedule 2.0 (WHODAS 2.0) to evaluate health conditions, disabilities across cultures, diseases in clinical intervention, and general population health. It has already been translated in 30 countries [4,5]. Thus, the multidimensional assessment is comprehensive and can be easily used to measure AP [4]. WHODAS 2.0 provides a standardized and generic method for measuring health and disability.

The government in Taiwan promotes the health of citizens and provides comprehensive coverage of free preventive health services, such as flu vaccinations, health examinations, and cancer screening [6]. In 2015, the Taiwan Longitudinal Study on Aging reported that 41.1% of women received Pap smear screening, whereas 53.3% of the middle-aged people in the general population underwent a check-up [7]. However, people with disabilities displayed less use of preventive health services, such as Pap smears (7.74–28.5%) [8,9] and breast screening (4.32–8.79%) [10,11]. To date, past studies on preventive health services have focused on associated factors, such as demographics, disease, and disability type for specific preventive health services [8,11], and no study has yet focused on overall preventive health services and the relationship with AP. Evidence has shown that preventive health services can detect or even prevent serious diseases and medical problems [12]. However, people with disabilities are less likely than the general population to use preventive health services, such as immunization; breast, cervical, and cancer screening; and oral health and general health examinations [13]. Given the disadvantages that people with disabilities face in using health-preventive health services and the important role that AP plays in health, it is critical to address AP performance among people with disabilities.

Therefore, providing a clear picture of the factors associated with the use of preventive health services including AP and other demographic concerns is essential. In addition, examining differences in the uses of preventive health services for people with disabilities will inform policymakers in designing concrete proposals and highlight the disparity in the use of preventive health services that should be considered in policy revisions. Therefore, the study aimed to elucidate the disparity in the use of preventive health services and identify the factors associated with the use of preventive health services.

## 2. Materials and Methods

To examine the disparity in the use of preventive health services for people with disabilities, and to realize predictions of preventive health service use and other associated factors, a cross-sectional survey was administered in Hualien, a city in east Taiwan.

### 2.1. Participants

The study samples were selected by stratified proportional sampling according to sex, age, and severity of disability in Taiwan. The sampling frame was selected according to a current register from the Social Affairs Department of Taiwan, updated in 2018 [14]. No statistical differences were found in sex, age, or severity of disability between the populations and samples.

There were 742 participants who fitted the following inclusion criteria: were (1) over 40 years old, (2) resident in Hualien city, (3) held a disability certification in Taiwan, and (4) willing to participate in the study. The data were collected through face-to-face interviews, and the participant information was provided by the people-with-disability database for Hualien. The interviewers completed professional training courses prior to the interviews. All of them were professional interviewers, with an average of 5 years of experience. The study approached 1084 people with disabilities. However, 342 participants were unavailable for interviews due to death (n = 25; 7.3%), moving to other cities (n = 71; 21.6%), or refusal to participate (n = 100; 29.5%). Others could not be reached through phone calls and in person (n = 146; 42.7%). The response rate was 68.45%. Every participant signed a consent form, and the research was approved by the Buddhist Tzu Chi General Hospital Research Ethics Committee (IRB 109-203-B).

### 2.2. Instruments

The 12-item version of WHODAS 2.0 was applied to measure AP in daily life over the previous 30 days [4]. AP represents the extent of the restriction of participation in daily life, and the qualifier of “performance” is described as what individuals do in their current environment. Because the current environment always includes an overall societal context, performance can also be understood as “involvement in a life situation” or “the lived experience” of people in their actual context.

AP comprises several concepts of functioning, such as (1) cognition, (2) getting around, (3) self-care, (4) getting along with people, (5) life activities, and (6) participation. The responses to each item ranged from 0 to 4 (0 = no difficulty, 1 = mild difficulty, 2 = moderate difficulty, 3 = severe difficulty, and 4 = extreme difficulty). The summary scores were calculated using the WHODAS 2.0 manual, and the scoring method comprises the addition of the scores for the 12 items, dividing the result by the maximum possible total (i.e., 48), and then multiplying by 100. The summary scores ranged from 0 to 100, with a higher score indicating higher limitations in daily life [4]. For the reliability and validity of AP, past studies have shown great internal consistency, test–retest consistency, and convergent and divergent validity [5,15,16,17]. The AP instruments’ reliability for people with disabilities in the present study showed great internal consistency (Cronbach alpha: 0.90).

The following data were collected on social demographics: gender, age, level of education (elementary and junior high, and senior high and above), and having caregivers (i.e., spouses, children and daughters-in-law, or foreign workers). Furthermore, data were collected on the use of any of the following preventive health services in the past 12 months: health examinations, flu vaccinations, cancer screening (colon or oral cancer), Pap smears, and breast screening. The males commonly used zero to three services, whereas the females used zero to five. Zero is defined as the non-use of preventive health services, whereas one to five is defined as the use of preventive health services in the present study. Monthly income was categorized as general, middle and low income. General income means income above the minimum living expense cutoff of 7750 USD/year, and low income, less than 5277 USD/year, based on government regulations [18]. Perceived health was evaluated as “not very good”, “not good”, “normal”, “good”, and “very good”. Disability severity was evaluated by medical physicians as mild, moderate, severe, or extremely severe [19].

The data were analyzed with the IBM SPSS 20.0 software, and the significance level for p-values was set at 0.01. Descriptive analysis was used to determine the distributions of the participant’s characteristics, and logistic regression was utilized to predict the associated factors of preventive health services and control for social demographics variables.

## 3. Results

The 742 participants were mostly male, and the average age was 65.19 years old. Most of the participants had an elementary or junior high school education level. Most responded as having no caregiver (58.5%) or having a spouse (22.5%), and around 84.6% had general income status (>7750 USD/year). The disability severity for most of the participants was mild (37.7 %), with many using preventive health services (70.2%). The perceived health for most of the participants was normal (61.2%). Furthermore, significant differences were found between the use and non-use of preventive health services in the abovementioned variables except AP and age (*p* < 0.01). The other social demographics were similar in the two groups. In terms of the AP scores, the study found that they were higher for the non-use (28.88 ± 32.11) than the use (20.85 ± 24.80) of preventive health services (Table 1).

For preventive health service use, we found that most of the participants were using one preventive health service (32.7%) or not using preventive health services (29.8%). After the investigation of the AP differences between the people who used or did not use preventive health services, the preventive health services were further divided into health examinations, flu vaccinations, cancer screening, Pap smears, and breast screening. People with disabilities showed the least use of all the preventive health services, especially cancer screening (15.9%). An examination of the AP scores for the use of preventive health services revealed that participants who used all the types of preventive health services had fewer limitations in daily life compared with those who did not use preventive health services. Participants who received health examinations, cancer screenings, Pap smears, and breast screening had significantly fewer limitations than those who did not use these services (*p* < 0.01) (Table 2).

Table 2 shows the distributions of people with disabilities who used and did not use preventive health services. Logistic regression was applied, and the covariates were controlled to test all the factor relationships for preventive health services. Those having caregivers of spouses (OR = 1.74) compared to the group without caregivers, those with perceived good health (OR = 1.26) compared to the group with perceived poor health, and less limitation of AP in daily life (OR = 0.99) were significantly associated with the use of preventive services (*p* < 0.01). Therefore, the participants who used each type of preventive service were considered separately to determine the predicted factors for each group. For specific preventive health services, we could see that older adults (OR = 1.79) compared to middle-aged (40–64 years) adults, less limitation of AP in daily life (OR = 0.98), and living in a rural area (OR = 0.39) compared to an urban area were significantly associated with the use of health examination services (*p* < 0.01). Older adults (OR = 2.32) compared to middle-aged (40–64 years) adults, those having caregivers of spouses (OR = 1.86) compared to the group without caregivers, less limitation of AP in daily life (OR = 0.99), and living in a rural area (OR = 0.58) compared to an urban area were significantly associated with the use of flu vaccinations (*p* < 0.01). For cancer screening, older adults (OR = 0.59) compared to middle-aged (40–64 years) adults, being male (OR = 0.49) compared to female, and a severe disability severity compared to the group of mild disability were significantly associated with the non-use of cancer screening (*p* < 0.01). In terms of preventive health services for females, having good perceived health was significantly associated with the use of breast screening services (OR = 6.12) and Pap smears (OR = 4.67). However, the study found a significant association between having children as caregivers compared to the group without caregivers, and the non-use of Pap smears (OR = 0.14) and breast screening services (OR = 0.08) (*p* < 0.01) (Table 3).

## 4. Discussion

To the best of our knowledge, the study is the first to examine the disparity in the use of preventive health services, such as health examinations, flu vaccinations, cancer screening, Pap smears, and breast screening, by focusing on associated factors for people with disabilities. The findings provide evidence that people with disabilities use fewer preventive health services, whereas those with less limitation of AP in life, perceived good health, and spouses as caregivers were deemed to utilize more preventive health services. Furthermore, the study provides a clear picture of the factors associated with the use of preventive health services.

### 4.1. Use of Preventive Health Services among People with Disabilities

A comparison with a previous survey on disability conducted in the same area indicated that the rate of health examinations improved from 34.4% in 2014 to 47.3% in the present study. A possible reason for this improvement is that government agencies have developed localized strategies to urge people to receive preventive health services to improve health. Such efforts have included establishing medical stations in remote areas and providing routine clinic services, outpatient medical services at night, preventive care, and health education, among other services [20]. Furthermore, government agencies need to provide specialized outpatient care for people with disabilities and set up mobile health clinics in villages to promote the direct delivery of such services to people with disabilities [20].

### 4.2. AP Factors Associated with the Use of Specific Preventive Health Services

The results correspond to the development of health policies that aim to promote to people the use of preventive health services. The study is the first to examine the relationship between AP performance and the use of preventive health services. The results indicate that better AP was associated with the use of preventive health services (OR = 0.99, *p* < 0.01). That is, patients who had lower daily life limitations in the study tended to use preventive health services (*p* < 0.01). Furthermore, previous studies have presented evidence that preventive health services can reduce costs and mortality and are correlated with disability severity but unrelated to the direct measurement of functioning [21,22].

By further categorizing preventive health services into specific types, the study found that less limitation of AP was significantly associated with the use of health examinations and flu vaccinations. In other words, people with disabilities with high levels of functioning seem to use preventive health services more frequently than those with low levels of functioning. In this regard, previous studies demonstrated that people with disabilities are less likely to use preventive health services due to a lack of access to transportation [23]. Therefore, promoting the use of preventive health services among people with disabilities to reduce disparities in health status became an important issue in Taiwan. In the previous year, the Taiwanese government provided government-funded flu vaccines for high-risk populations to increase the coverage of services, such as individuals aged 50 years and above, people with disabilities living in institutions or communities, and those with rare diseases or major illnesses. To reduce inconvenience, the Taiwanese government also set up stations for flu vaccination in communities and provided services delivering flu vaccinations to homes or institutions directly [24]. Specifically, to reduce inconvenience in terms of transportation for people with disabilities, the government dispersed mobile health clinics that directly administer preventive health services in villages. However, there were no significant differences in breast screening and Pap smears; this is probably due to, as past studies have pointed out, the difficulties in receiving medical treatment for people with disabilities, such as a lack of adjusted medical equipment, or lack of assistive devices in community screening, in addition to a lack of understanding and knowledge among health professionals with regard to people with disabilities [25]. The results of the present study elucidate that the rate of the use of preventive services for people with disabilities needs improvement because the majority use approximately one service. Therefore, formulating strategies to overcome the barriers to the use of preventive services for people with disabilities is a critical issue that the government should consider. In addition, medical staff should accumulate knowledge and consensus regarding the needs of people with disabilities.

### 4.3. The Relationship between Caregivers, Perceived Health, and Preventive Health Services

In the present study, people with disabilities with spouses tended to use preventive health services, which is consistent with the observations of previous studies [26]. Another interesting result is that if children acted as caregivers, then women with disabilities were less likely to utilize Pap smears and breast screening services. A possible reason is that the prevalent culture for women in Asia is to take responsibility in caring for the family. Thus, they are less likely to allocate time for themselves. Previous studies reported that women in Asian countries are less likely to recognize the symptoms of a health problem and do not treat it as serious or warranting medical help [27]. A national survey report for the needs of people with disabilities in Taiwan demonstrated that the main difficulties experienced in seeking medical help were a lack of transportation and being unaccompanied [23]. Although the Taiwanese government provides services, such as home care and personal assistance, to enable people with disabilities to seek medical services, the need to increase the health consciousness of people with disabilities and to create a friendly physical and social environment remains urgent for health agencies in ensuring understanding for people with disabilities [28]. Previous research found that perceived good health is associated with the use of preventive health services among people with disabilities. In other words, perceived health was a reliable indicator correlated with the use of preventive health services for the general population and in the present study [29,30]. People with disabilities exhibited correlations with high levels of disability severity and with more limitations, which is consistent with the findings of previous studies [8,11]. However, the values explaining the variance (R^2^) in the models were low. Thus, determining other unidentified variables, such as transportation, accompaniment needs, physical and social environment support, and medical staff attitudes, is also necessary for further examination. The findings of the present study point to the existing health disparities. In this regard, reducing the gap between people with disabilities and the general population is important. Furthermore, the Convention on the Rights of Persons with Disabilities (CRPD) aims to reduce inequalities across countries and improve access to health services and programs for people with disabilities [28].

## 5. Conclusions

The study bears its share of limitations. First, the participants were recruited from one county in Taiwan. Thus, although no statistical differences were noted in terms of gender, age, and severity of disability between the populations and samples in the county, the external validity may be limited when considering Taiwan in general. Moreover, the study may have been biased towards persons who have used preventatives, given the relatively high proportion of persons who declined to take part in the study. Nevertheless, the present study provides valuable evidence of the distribution of the use of preventive health services, such as health examinations, flu vaccinations, cancer screening, Pap smears, and breast screening for people with disabilities, as well as the association of a high level of AP limitation with the non-use of preventive health services. In addition, age, having spouses as caregivers, perceived good health, and less limitation of AP functioning were beneficial for the use of preventive health services for people with disabilities (*p* < 0.01). For women with disabilities, the results indicate a significant association between having children as caregivers and the non-use of Pap smears and breast screening. Twelve items for AP were derived from WHODAS 2.0 as a tool for evaluating individual functioning within a short time; using the full version will enable future studies to examine the relationships between subdomains of AP and the use of preventive services in detail. Nevertheless, the study presents a clear picture of the factors associated with the use of preventive health services.

## Figures and Tables

**Table 1:** Social demographic characteristics of people with disabilities in terms of use and non-use of preventive health services.

Variable		All Participants (n = 742)		A. Use of Preventive Health Services (n = 521)		B. Non-Use of Preventive Health Services (n = 221)		*p* Value of Difference between A and B
		n	%	n	%	n	%	
Gender	Male	411	55.4	287	55.1	124	56.1	0.80
	Female	331	44.6	234	44.9	97	43.9	
Age		65.19 ± 13.85		66.68 ± 13.18		62.05 ± 14.69		0.001 **
	40–64	386	52.0	250	48.0	136	61.5	0.001 **
	65 and above	356	48.0	271	52.0	85	38.5	
Education	Elementary and junior high	475	64.0	337	64.7	138	62.4	0.56
	Senior high and above	267	36.0	184	35.3	83	37.6	
Town	Rural	88	11.9	63	12.1	25	11.3	0.76
	Urban	654	88.1	458	87.9	196	88.7	
Caregiver	None	434	58.5	310	59.5	124	56.1	0.55
	Spouse	167	22.5	119	22.8	48	21.7	
	Children	85	11.5	56	10.7	29	13.1	
	Foreign worker	56	7.5	36	6.9	20	9.0	
Monthly income	General (>7750 USD/year)	598	84.6	415	83.5	183	87.1	0.44
	Middle (≤7750 USD/year)	64	9.1	49	9.9	15	7.1	
	Low (≤5277 USD/year)	45	6.4	33	6.6	12	5.7	
Disability severity	Mild	280	37.7	202	38.8	78	35.3	0.73
	Moderate	238	32.1	162	31.1	76	34.4	
	Severe	143	19.3	102	19.6	41	18.6	
	Extremely severe	81	10.9	55	10.6	25	11.8	
Perceived health	Not very good	21	2.8	12	2.3	9	4.1	0.49
	Not good	168	22.6	114	21.9	54	24.4	
	Normal	454	61.2	322	61.8	132	59.7	
	Good	97	13.1	72	13.8	25	11.3	
	Very good	2	0.3	1	0.2	1	0.5	
AP		23.44 ± 27.60		20.85 ± 24.80		28.88 ± 32.11		<0.001 ***

Note. Statistics are shown as mean (±SD) for continuous variables and number (percentage) for categorical variables. Significance levels: ** *p* < 0.01; *** *p* < 0.001. AP = activity and participation.

**Table 2:** Distribution of the use of preventive health services and AP scores for people with disabilities.

Numbers of Preventive Health Services Used	Male	Female		All
	Number (Percentage)	Number (Percentage)		Number (Percentage)
0	124 (30.2)	97 (29.3)		221 (29.8)
1	135 (32.8)	108 (32.6)		243 (32.7)
2	124 (30.2)	70 (21.1)		194 (26.1)
3	28 (6.8)	32(9.7)		60 (8.1)
4	-	21 (6.3)		21 (2.8)
5	-	3 (0.9)		3 (0.4)
All	411	331		742
Specific preventive health services	A. Use of preventive health services (n = 521)		B. Non-use of preventive health services (n = 221)	
	Number (Percentage)	AP Score	Number (Percentage)	AP Score
Health examination	351 (47.3)	20.09 ± 24.54	391 (52.7)	26.11 ± 29.59 **
Flu vaccination	302 (40.7)	21.43 ± 25.29	440 (59.3)	24.63 ± 28.86
Cancer screening	118 (15.9)	16.30 ± 23.14	624 (84.1)	24.73 ± 28.16 **
Pap smear (n = 331)	75 (22.7)	12.43 ± 18.00	256 (77.3.)	28.58 ± 27.96 ***
Breast screening (n = 331)	64 (19.3)	17.16 ± 20.41	267 (80.7)	26.84 ± 27.97 **

Note. Statistics are shown as number (percentage) for categorical variables. Significance levels of differences between groups: ** *p* < 0.01; *** *p* < 0.001.

**Table 3:** Factors associated with the use of preventive health services for people with disabilities.

Variable		Preventive Services	Health Examination	Flu Vaccination	Cancer Screening	Breast Screening	Pap Smear
		OR (95% CI)	OR (95% CI)	OR (95% CI)	OR (95% CI)	OR (95% CI)	OR (95% CI)
Age (ref: 40–64 years old)		1.38 (0.94, 2.00)	1.79 (1.22, 2.61) **	2.32([1.57, 3.42) ***	0.59 (0.35, 0.98) *	0.65 (0.31, 1.35)	0.72 (0.36, 1.43)
Gender (ref: male)		1.29 (0.92, 1.82)	0.79 (0.56, 1.11)	0.81 (0.57, 1.16)	0.49 (0.30, 0.80) **	-	-
Education (ref: elementary and junior high	Senior high and above	1.18 (0.82, 1.70)	0.99 (0.69, 1.44)	1.07 (0.73, 1.56)	1.04 (0.63, 1.68)	0.82 (0.37, 1.80)	0.96 (0.46, 1.98)
Township (ref: rural)	Urban	0.83 (0.51, 1.34)	0.39 (0.24, 0.65) ***	0.58 (0.35, 0.96) *	0.82 (0.43, 1.56)	0.62 (0.24, 1.58)	0.71 (0.28, 1.80)
Caregiver	Spouse	1.74 (1.15, 2.63)**	1.23 (0.81, 1.83)	1.86 (1.22, 2.82) **	1.33 (0.78, 2.18)	1.31 (0.60, 2.86)	1.31 (0.62, 2.77)
(ref: none)	Children	0.97 (0.56, 1.68)	0.92 (0.52, 1.61)	1.56 (0.89, 2.74)	0.90 (0.37, 2.23)	0.08 (0.02, 0.38) **	0.14 (0.04, 0.51) **
	Foreign worker	0.86 (0.43, 1.68)	1.59 (0.80, 3.15)	1.23 (0.60, 2.48)	0.95 (0.29, 3.09)	-	0.10 (0.01, 0.81) *
Monthly income (ref: low and middle income)	Normal (>7750 USD/year)	0.70 (0.42, 1.17)	0.91 (0.54, 1.53])	0.79 (0.46, 1.36	0.85 (0.43, 1.70)	0.86 (0.28, 2.67)	0.79 (0.28, 2.27)
Disability severity	Moderate	0.83 (0.83, 1.22)	0.76 (0.52, 1.12)	1.15 (0.78, 1.71)	0.58 (0.34, 0.98) *	0.60 (0.28, 1.28)	0.86 (0.43, 1.74)
(ref: mild)	Severe	0.79 (0.48, 1.29)	0.82 (0.49, 1.35])	0.99 (0.59, 1.67)	0.46 (0.22, 0.95) *	0.37 (0.12, 1.13)	0.68 (0.25, 1.85)
	Extremely severe	0.72 (0.40, 1.3)	1.12 (0.62, 2.02)	1.23 (0.67, 2.23)	0.30 (0.11, 0.80) *	0.39 (0.10, 1.57)	0.36 (0.09, 1.40)
Perceived health ^a^	Normal	1.86 (1.04, 3.3)	0.88 (0.59, 1.32)	1.02 (0.67, 1.55)	1.29 (0.70, 2.37)	0.98 (0.44, 2.18)	1.12 (0.53, 2.40)
(ref: not good)	Good	1.26 (0.84, 1.87) *	0.93 (0.46, 1.48)	1.20 (0.66, 2.18)	1.74 (0.79, 3.85)	6.12 (1.92, 19.48)	4.67 (1.55, 14.06) **
AP		0.99 (0.98, 0.99) *	0.98 (0.98, 0.99) **	0.99 (0.98, 0.99)	0.99 (0.98, 1.01)	1.00 (0.99, 1.02)	0.99 (0.97, 1.00)
Nagelkerke R^2^		0.08	0.08	0.09	0.10	0.31	0.28

Note. Significance levels: * *p* < 0.05; ** *p* < 0.01; *** *p* < 0.001. ^a^ In terms of perceived health, the variables “not very good” and “not good” were merged and renamed “not good”, whereas the variables “good” and “very good” were merged and renamed “good” due to the small sample size.

## Data Availability

Restrictions apply to the availability of these data. Data was obtained from Hualien Social Affairs Department and are available with the permission of Hualien Social Affairs Department.

## References

[1] WHO (2019). World Health Statistics 2019: Monitoring Health for the SDGs, Sustainable Development Goals.

[2] World Health Organization (2015). WHO Global Disability Action Plan 2014–2021.

[3] World Health Organization (2001). International Classification of Functioning, Disability and Health (ICF).

[4] Üstün T.B., Kostanjsek N., Chatterji S., Rehm J. (2010). Measuring Health and Disability: Manual for WHO Disability Assessment Schedule WHODAS 2.0.

[5] Saltychev M., Katajapuu N., Bärlund E., Laimi K. (2019). Psychometric properties of 12-item self-administered World Health Organization disability assessment schedule 2.0 (WHODAS 2.0) among general population and people with non-acute physical causes of disability—systematic review. Disabil. Rehabil..

[6] Health Promotion Administration (2015). The Manual of Preventive Services for Adults in Taiwan.

[7] Health Promotion Administration (2018). 2015 Taiwan Longitudinal Study on Aging Survey Report.

[8] Huang K.-H., Tsai W.-C., Kung P.-T. (2012). The use of Pap smear and its influencing factors among women with disabilities in Taiwan. Res. Dev. Disabil..

[9] Lin J.-D., Chen S.-F., Lin L.-P., Sung C.-L. (2011). Self-reports of Pap smear screening in women with physical disabilities. Res. Dev. Disabil..

[10] Lai H.-T., Kung P.-T., Tsai W.-C. (2014). Factors influencing the mammography utilization among Taiwanese women with intellectual disabilities, a nationwide population-based study. Res. Dev. Disabil..

[11] Yen S.-M., Kung P.-T., Tsai W.-C. (2015). Mammography usage with relevant factors among women with mental disabilities in Taiwan: A nationwide population-based study. Res. Dev. Disabil..

[12] U.S. Preventive Services Task Force, Agency for Healthcare Research and Quality (2006). The Guide to Clinical Preventive Services: Recommendations of the U.S. Preventive Services Tack Force.

[13] World Health Organization (2011). World Report on Disability.

[14] Hualien Social Affairs Department (2018). The Population in Hualien County.

[15] Silveira C., Souza R.T., Costa M.L., Parpinelli M.A., Pacagnella R.C., Ferreira E.C., Mayrink J., Guida J.P., Sousa M.H., Say L. (2018). Validation of the WHO Disability Assessment Schedule (WHODAS 2.0) 12-item tool against the 36-item version for measuring functioning and disability associated with pregnancy and history of severe maternal morbidity. Int. J. Gynaecol. Obs..

[16] Younus M.I., Wang D.M., Yu F.F., Fang H., Guo X. (2017). Reliability and validity of the 12-item WHODAS 2.0 in patients with Kashin-Beck disease. Rheumatol. Int..

[17] Axelsson E., Lindsäter E., Ljótsson B., Andersson E., Hedman-Lagerlöf E. (2017). The 12-item Self-Report World Health Organization Disability Assessment Schedule (WHODAS) 2.0 Administered Via the Internet to Individuals with Anxiety and Stress Disorders: A Psychometric Investigation Based on Data From Two Clinical Trials. JMIR Ment. Health.

[18] Hualien Social Affairs Department (2020). Subsidy for Low-Income Family.

[19] Yen C.F., Hwang A.W., Liou T.H., Chiu T.Y., Hsu H.Y., Chi W.C., Wu T.F., Chang B.S., Lu S.J., Liao H.F. (2014). Validity and reliability of the Functioning Disability Evaluation Scale-Adult Version based on the WHODAS 2.0--36 items. J. Formos. Med. Assoc. Taiwan Yi Zhi.

[20] Ministry of Health and Welfare (2014). Distance to Quality-Eight Strategies to Upgrade Health Care in Taiwan’s Remote Area.

[21] Farley T.A., Dalal M.A., Mostashari F., Frieden T.R. (2010). Deaths Preventable in the U.S. by Improvements in Use of Clinical Preventive Services. Am. J. Prev. Med..

[22] Maciosek M.V., Coffield A.B., Edwards N.M., Flottemesch T.J., Goodman M.J., Solberg L.I. (2006). Priorities among effective clinical preventive services: Results of a systematic review and analysis. Am. J. Prev. Med..

[23] Ministry of Health and Welfare (2018). Report of Disabled People’s Living Condition and Demand Survey.

[24] Centers for Disease Control and Prevention (2020). The Project of Flu Vaccination in 2020.

[25] Lin Y.-C., Chang H.H. (2018). When “Non-Standard Patients” Encounter the Medical Professional System: A Qualitative Analysis of Medical Experiences among People with Disabilities. NTU Soc. Work Rev..

[26] Blumberg S.J., Vahratian A., Blumberg J.H. (2014). Marriage, cohabitation, and men’s use of preventive health care services. NCHS Data Brief.

[27] WHO (2000). Women in SE Asia: A Health Profile.

[28] United Nations (2015). Convention on the Rights of Persons with Disabilities.

[29] Palladino R., Tayu Lee J., Ashworth M., Triassi M., Millett C. (2016). Associations between multimorbidity, healthcare utilisation and health status: Evidence from 16 European countries. Age Ageing.

[30] Spiers N., Jagger C., Clarke M. (1996). Physical Function and Perceived Health: Cohort Differences and Interrelationships in Older People. J. Gerontol. Ser. B.

